# Absolute Eosinophil Count Predicts Intensive Care Unit Transfer Among Elderly COVID-19 Patients From General Isolation Wards

**DOI:** 10.3389/fmed.2020.585222

**Published:** 2020-11-04

**Authors:** Jinjin Huang, Zhicheng Zhang, Shunfang Liu, Chen Gong, Liping Chen, Guo Ai, Xiaodong Zhu, Chunli Zhang, Dengju Li

**Affiliations:** ^1^Department of Hematology, Tongji Hospital, Tongji Medical College, Huazhong University of Science and Technology, Wuhan, China; ^2^Department of Gastroenterology, Tongji Hospital, Tongji Medical College, Huazhong University of Science and Technology, Wuhan, China; ^3^Department of Molecular and Medical Pharmacology, University of California, Los Angeles, CA, United States; ^4^Department of Oncology, Tongji Hospital, Tongji Medical College, Huazhong University of Science and Technology, Wuhan, China; ^5^Department of Pediatrics, Tongji Hospital, Tongji Medical College, Huazhong University of Science and Technology, Wuhan, China; ^6^Department of Oncology, Huang Gang Central Hospital, Huanggang, China; ^7^Department of Endocrinology, Huang Gang Central Hospital, Huanggang, China

**Keywords:** COVID-19, eosinophils, prediction, severity, intensive care unit

## Abstract

**Objectives:** As of June 1, 2020, coronavirus disease 2019 (COVID-19) has caused a global pandemic and resulted in over 370,000 deaths worldwide. Early identification of COVID-19 patients who need to be admitted to the intensive care unit (ICU) helps to improve the outcomes. We aim to investigate whether absolute eosinophil count (AEC) can predict ICU transfer among elderly COVID-19 patients from general isolation wards.

**Methods:** A retrospective study of 94 elderly patients older than 60 years old with COVID-19 was conducted. We compared the basic clinical characteristics and levels of inflammation markers on admission to general isolation wards and the needs for ICU transfer between the eosinopenia (AEC on admission <20 cells/μl) and non-eosinopenia (AEC ≥20 cells/μl) groups.

**Results:** There was a significantly higher ICU transfer rate in the eosinopenia group than in the non-eosinopenia group (51 vs. 9%, *P* < 0.001). Multivariate analysis revealed that eosinopenia was associated with an increased risk of ICU transfer in elderly COVID-19 patients [adjusted odds ratio (OR) 6.12 (95% CI, 1.23–30.33), *P* = 0.027] after adjustment of age, lymphocyte count, neutrophil count, C-reactive protein (CRP), and ferritin levels. The eosinopenia group had higher levels of CRP, ferritin, and cytokines [interleukin-2 receptor (IL-2R), interleukin-6 (IL-6), interleukin-8 (IL-8), interleukin-10 (IL-10), and tumor necrosis factor-α (TNF-α)] than the non-eosinophil group (*P* < 0.001). The area under the curve of AEC on admission for predicting ICU transfer among elderly COVID-19 patients was 0.828 (95% CI, 0.732–0.923). The best cut-off value of AEC was 25 cells/μl with a sensitivity of 91% and a specificity of 71%, respectively.

**Conclusion:** Absolute eosinophil count on admission is a valid predictive marker for ICU transfer among elderly COVID-19 patients from general isolation wards and, therefore, can help case triage and optimize ICU utilization, especially for health care facilities with limited ICU capacity.

## Introduction

Coronavirus disease 2019 (COVID-19) caused by severe acute respiratory syndrome coronavirus 2 (SARS-CoV-2) infection has been spreading worldwide with over 6 million cases and more than 370,000 deaths by June 1, 2020 (1, 2). A study of 34,249 COVID-19 patients in Spain found that patients aged ≥60 years accounted for 68% of hospitalizations, 70% of intensive care unit (ICU) admissions, and 94% of mortalities (3). It is implied that elderly COVID-19 patients are more likely to have critical progression, which poses a major challenge in health care facilities with limited ICU capacity (4, 5).

Currently, early discrimination of elderly COVID-19 patients with ICU requirement remains challenging in clinics. Recently, the traditional inflammation markers, such as C-reactive protein (CRP) (6), ferritin (7), and cytokines (8), are considered as predictive markers for COVID-19 severity. However, these inflammation indicators are usually expensive and time-consuming (9). Thus, a cost-effective, convenient, and fast predictive marker for severe elderly patients with COVID-19 is highly demanded during this pandemic.

Eosinophils are multifunctional cells involved in inflammatory response triggered by diverse stimuli (10). It is already known that acute bacterial infection would result in eosinopenia (11). Some studies have found that eosinopenia on ICU admission could be a useful biomarker for children mortality (12), 28-day mortality (13), unexpected ICU re-admission, and post-ICU mortality (14). However, there is no evidence showing that eosinopenia and ICU admission of COVID-19 are closely relevant. Therefore, our study aims to evaluate the value of absolute eosinophil count (AEC) on admission as a predictive marker for ICU transfer among elderly COVID-19 patients from general isolation wards.

## Methods

### Study Design

This retrospective study included COVID-19 elderly patients aged ≥60 years from four general wards at the Sino-French New City Branch of Tongji Hospital in Wuhan between February 10, 2020 and February 20, 2020. Exclusion criteria included: (1) patients who had taken corticosteroids before admission, (2) patients who died within 24 h after admission, and (3) patients with a history of allergies and parasitic infections. The protocol of our study was approved by the Tongji Hospital Ethics Committee.

### Criteria of ICU Transfer for COVID-19

The criteria of ICU transfer for elderly COVID-19 patients included: (1) severe dyspnea that responds inadequately to initial emergency therapy, (2) changes in mental status (confusion, lethargy, coma, etc.), (3) persistent or worsening hypoxemia and/or severely worsening respiratory acidosis despite supplemental oxygen and non-invasive ventilation, and (4) the need for invasive mechanical ventilation and/or hemodynamic instability.

### Data Collection

Clinical data were extracted from electronic medical records, including age, sex, comorbidities, and blood routine and blood biochemistry values on admission. Records of inflammation indicators, such as CRP, ferritin, and cytokines [interleukin-2 receptor (IL-2R), interleukin-6 (IL-6), interleukin-8 (IL-8), interleukin-10 (IL-10), and tumor necrosis factor-α (TNF-α)], were also collected on admission.

### Laboratory Procedures

The levels of IL-2R, IL-8, IL-10, and TNF-α in serum were measured according to an automatic procedure of a solid-phase two-site chemiluminescent immunometric assay *via* the IMMULITE 1000 Analyzer (Siemens, Munich, Germany). The level of IL-6 was measured using the electrochemiluminescence method (Roche Diagnostics, Basel, Switzerland).

### AEC Profiling

Peripheral blood samples of our enrolled patients were assayed for full blood panel count including AEC on the Sysmex XS-800i (Sysmex Shanghai Ltd., Shanghai, China) as per the manufacturer's protocol. In accordance with the normal range of AEC in the Chinese population (15), we defined eosinopenia as AEC <20 cells/μl and non-eosinopenia as AEC ≥20 cells/μl. According to AEC on admission, the patients were divided into the eosinopenia group and the non-eosinopenia group.

### Statistical Analysis

Continuous variables were described as means (±standard deviation, SD) or medians (interquartile ranges, IQRs) according to their distributions checked by Kolmogorov–Smirnov-test. Student's *t*-test or Mann–Whitney U-test was used to compare continuous variables. Categorical variables were compared by chi-square or Fisher's exact-tests. Variables with *P*-value <0.10 on univariate analysis were tested in multivariate analysis. The odds ratio (OR) along with the 95% confidence interval (CI) was reported. The receiver operating characteristics (ROC) curve and the area under the curve (AUC) were calculated for AEC in predicting ICU transfer of patients on admission. The best eosinophil cut-off value was decided depending on the Youden index. All statistical analyses were conducted using GraphPad Prism 6 (GraphPad Software, San Diego, CA, USA) 16.0 and STATA 15 (StataCorp, College Station, TX, USA). A two-tailed *P*-value <0.05 was considered to be statistically significant.

## Results

### Patient Characteristics

**Figure 1:**
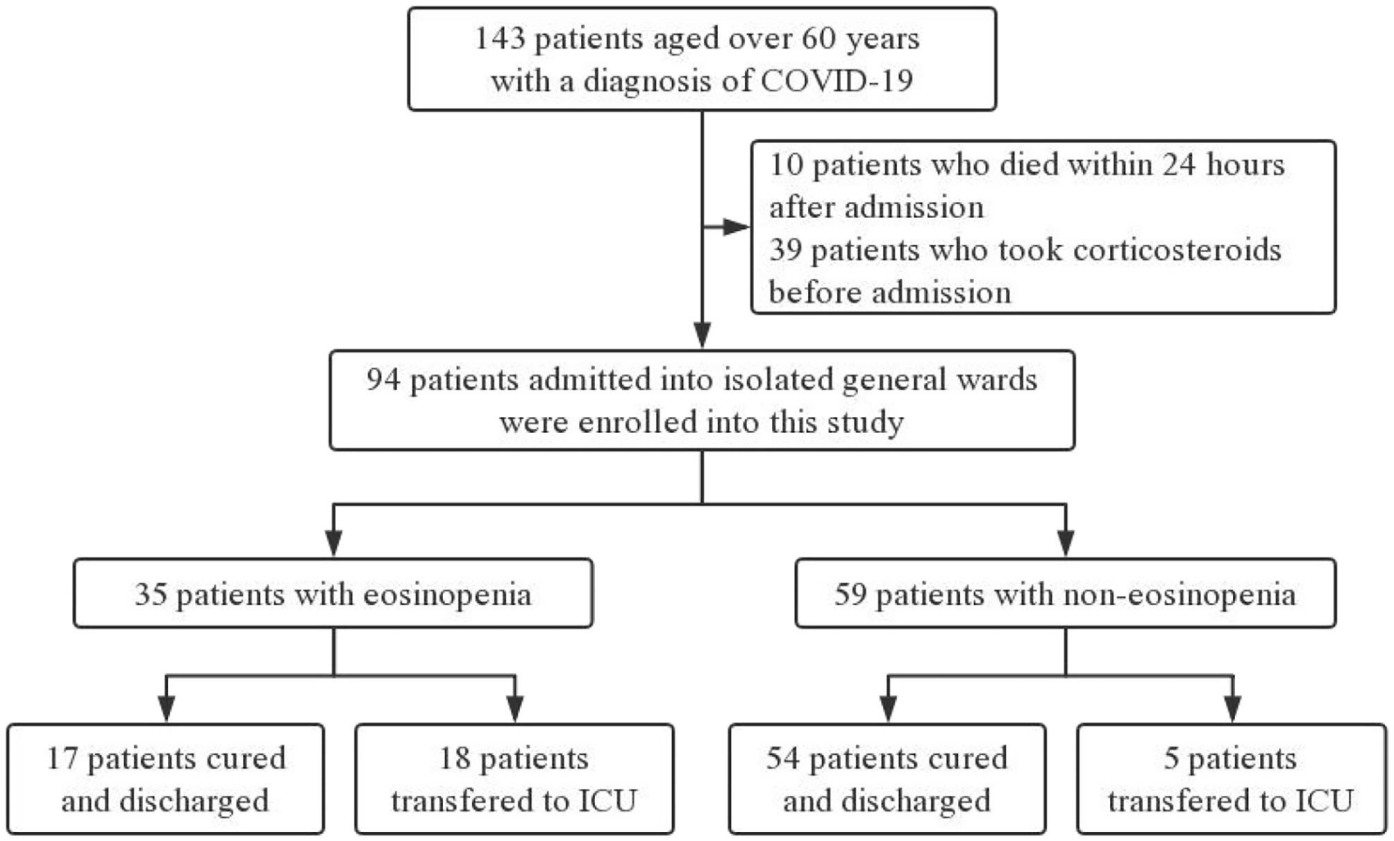
Flowchart of participants enrollment.

Electronic medical records of 143 COVID-19 patients older than 60 years old were collected by two physicians from four general isolation wards. After excluding 39 patients who had taken corticosteroids before admission and 10 patients who died within 24 h after admission, 94 participants were enrolled for further analysis (Figure 1).

The median age of the 94 patients was 70 years (IQR 65–76 years), ranging from 60 to 88 years (60–69 years, 47%; 70–79 years, 38%; >80 years, 15%). There were more males than females in this cohort (56 vs. 44%). Comorbidities were presented in nearly three quarters of these elderly patients, with hypertension being the most common comorbidity, followed by diabetes and coronary heart disease. There were no significant differences in age, sex, and comorbidity between the eosinopenia group (35 patients) and the non-eosinopenia group (59 patients) (*P* > 0.05; Table 1). However, the levels of lymphocyte count, platelet count, aspartate aminotransferase, hypersensitive cardiac troponin I, and serum sodium were significantly different between the two groups (*P* < 0.001; Table 2).

**Table 1:** Basic characteristics of elderly COVID-19 patients on admission (*n* = 94).

**Characteristics**	**Total**	**Eosinopenia**	**Non-eosinopenia**	***P***
Patients	94	35 (37%)	59 (63%)	–
Age, years	70 (64–76)	68 (64–78)	70 (66–75)	0.919
60–69	44 (47%)	18	26	
70–79	36 (38%)	10	26	0.864
≥80	14 (15%)	7	7	
Sex	–	–	–	0.752
Male	53 (56%)	19	34	–
Female	41 (44%)	16	25	–
Comorbidities	66 (70%)	–	–	–
Diabetes	31 (33%)	11	20	0.806
Hypertension	43 (46%)	15	28	0.665
Coronary heart disease	13 (14%)	5	8	0.921
Thyroid disease	1 (1%)	0	1	–
Parkinson's disease	1 (1%)	0	1	–
Systemic lupus erythematosus	1 (1%)	0	1	–

*Data are presented as median (interquartile range) or as number (percentage). Student's t-test or Mann–Whitney U-test was used to compare continuous variables*.

**Table 2:** Laboratory findings of elderly COVID-19 patients on admission (*n* = 94).

**Characteristics**	**Total**	**Eosinopenia**	**Non-eosinopenia**	***P***
WBC, × 10^9^/L	5.72 (4.49–7.95)	5.87 (3.90–8.88)	5.74 (4.49–7.58)	0.614
Neutrophils count, × 10^9^/L	4.08 (2.86–6.18)	4.88 (2.91–7.68)	3.53 (2.80–5.82)	0.063
Lymphocytes count, × 10^9^/L	1.03 (0.68–1.39)	0.64 (0.50–0.90)	1.24 (0.89–1.63)	<0.001
Hemoglobin, g/L	124 (116–136)	129 (118–142)	121 (113–129)	0.012
Platelet count, × 10^9^/L	222 (154–302)	155 (137–224)	249 (186–342)	<0.001
PT, s	14.1 (13.5–14.7)	14.4 (13.5–15.1)	14.0 (13.5–14.5)	0.100
APTT, s	38.1 (35.4–42.1)	39.7 (36.4–42.3)	36.7 (35.4–41.1)	0.238
D-dimer, mg/L	0.93 (0.42–1.89)	1.16 (0.60–1.90)	0.69 (0.39–1.65)	0.043
ALT, U/L	22 (14–32)	24 (17–38)	21 (13–28)	0.095
AST, U/L	25 (19–35)	34 (25–49)	22 (19–28)	<0.001
Total bilirubin, mmol/L	9.1 (6.5–11.8)	11.2 (7.9–13.6)	8.1 (6.3–10.9)	0.013
Albumin, g/L	34.4 (31.1–38.5)	33.7 (30.8–38.0)	35.1 (31.7–39.1)	0.269
Hs-cTnI, pg/ml	6.7 (3.6–11.4)	11.9 (7.3–24.4)	4.9 (2.6–7.2)	<0.001
NT-proBNP, pg/ml	188 (91–374)	233 (105–885)	171 (74–289)	0.013
Potassium, mmol/L	4.23 (3.85–4.60)	4.34 (3.96–4.55)	4.21 (3.77–4.76)	0.955
Sodium, mmol/L	140 (137–143)	138 (135–141)	141 (139–143)	<0.001
Calcium, mmol/L	2.40 (2.33–2.46)	2.36 (2.27–2.48)	2.41 (2.36–2.46)	0.019
GFR, ml/min	82.3 (71.3–91.3)	80.3 (61.9–88.0)	84.2 (75.1–92.8)	0.060
Creatinine, μmol/L	69 (60–87)	78 (61–93)	68 (58–82)	0.054
Blood glucose, mmol/L	6.6 (5.7–8.5)	6.8 (6.0–9.4)	6.4 (5.3–8.1)	0.022

*Data are presented as median (interquartile range)*.

*P < 0.05 was considered significant*.

*PT, prothrombin time; APTT, activated partial thromboplastin time; GFR, glomerular filtration rate; WBC, white blood cell; ALT, alanine aminotransferase; AST, aspartate aminotransferase; Hs-cTnI, hypersensitive cardiac troponin I; NT-ProBNP, NT-ProB-Type natriuretic peptide*.

### Association Between Eosinopenia and ICU Transfer of Elderly COVID-19 Patients

The median duration from illness onset to hospital admission was 10 days (IQR 7–16 days) for all patients, with 7 days (IQR 5–12 days) for the eosinopenia group and 14 days (IQR 8–19 days) for the non-eosinopenia group, respectively. There was a significantly higher ICU transfer rate in the eosinopenia group than in the non-eosinopenia group (51 vs. 9%, *P* < 0.001; Table 3).

**Table 3:** Characteristics of elderly COVID-19 patients after illness onset (*n* = 94).

**Characteristics**	**Total**	**Eosinopenia**	**Non-eosinopenia**	***P***
Number of patients	94	35	59	–
From onset to admission (days)	10 (7–16)	7 (5–12)	14 (8–19)	<0.001
From general isolation wards to the ICU (number of patients)	23 (24%)	18 (51%)	5 (9%)	<0.001
From general isolation wards to the ICU (days)	3 (2–10)	5 (2–9)	3 (2–13)	0.927

*Data are presented as median (interquartile range) or as number (percentage). P < 0.05 was considered as statistically significant*.

*ICU, intensive care unit*.

In univariate analysis, a significant inverse association between eosinopenia and ICU transfer of elderly COVID-19 patients was revealed [crude OR 11.44 (95% CI, 3.69–35.43), *P* < 0.001; Table 3]. Furthermore, the inverse association remained significant [adjusted OR 6.12 (95% CI, 1.23–30.33), *P* = 0.027] by multivariate analysis after adjustment for age, lymphocyte count, neutrophil count, CRP, and ferritin levels (Table 4).

**Table 4:** Association between eosinopenia and ICU transfer.

	**Eosinopenia and ICU transfer**	***P***
Crude OR (95% CI)	11.44 (43.69–35.43)	<0.001
Adjusted OR (95% CI)	6.12 (1.23–30.33)	0.027

*Multiple logistic regression analysis was adjusted for the following variables: age, lymphocyte count, neutrophil count, CRP, and ferritin. P < 0.05 was considered as statistically significant*.

*ICU, intensive care unit; OR, odds ratio; CI, confidence interval; CRP, C-reactive protein*.

Surprisingly, 61% (14 out of 23) of patients transferred to the ICU demonstrated AEC at zero, whereas this phenomenon applied to only 17% (12 out of 71) of patients discharged from the general isolation wards (Table 5).

**Table 5:** Number of patients with AEC of zero and non-zero.

	**AEC of zero (number of patients)**	**AEC of non-zero (number of patients)**
Number of patients transferred to the ICU	14	9
Number of patients discharged from the general isolation wards	12	59

*AEC, absolute eosinophil count; ICU, intensive care unit*.

### Association Between Eosinopenia and Traditional Inflammation Indicators

**Figure 2:**
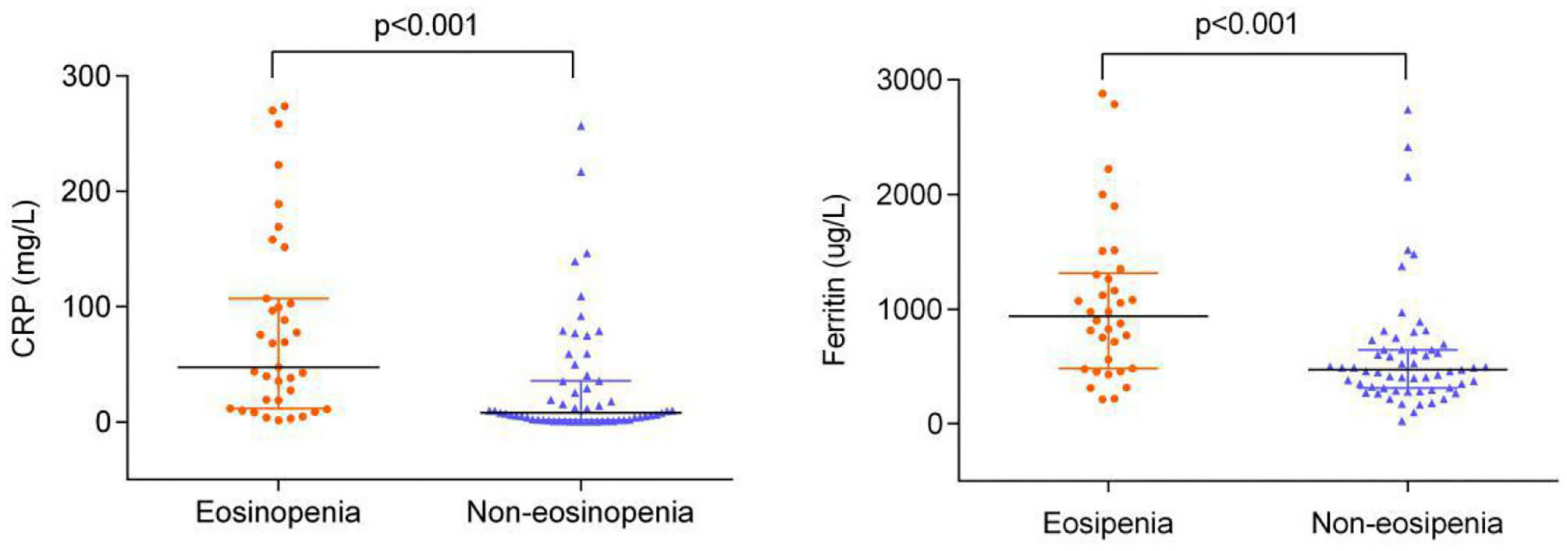
CRP and ferritin levels of elderly COVID-19 patients on admission (*n* = 94).

The levels of CRP and ferritin were much higher in the eosinopenia group than in the non-eosinopenia group with significant differences (*P* < 0.001; Figure 2). Additionally, 76 elderly patients in this cohort were tested for serum cytokines. We found that IL-2R, IL-6, IL-8, IL-10, and TNF-α levels were significantly higher in the eosinopenia group than in the non-eosinopenia group (*P* < 0.05; Table 6).

**Table 6:** Serum cytokine levels of patients (*n* = 76).

**Characteristics**	**Total**	**Eosinopenia**	**Non-eosinopenia**	***P***
IL-2R, U/ml	674 (418–918)	840 (615–1,106)	596.5 (385–835)	0.02
IL-6, pg/L	11.41 (3.96–39.18)	40.23 (14.82–80.33)	7.17 (2.87–14.33)	<0.001
IL-8, pg/L	11.9 (7.1–24.7)	13.9 (9.8–29.0)	10.3 (5.7–18.8)	0.045
IL-10, pg/L^*^	5.0 (5.0–7.7)	8.7 (5.8–13.9)	5.0 (5.0–5.0)	<0.001
TNF-α, pg/L^*^	8.3 (6.0–10.4)	9.8 (8.0–12.9)	7.1 (5.0–9.2)	0.01

* *The concentrations of TNF-α and IL-10 were unable to be detected below 5 pg/L. The values below 5 pg/L were replaced by 5 pg/L in statistical analysis*.

*Data are presented as median (interquartile range). P < 0.05 was considered as statistically significant*.

*IL-2R, interleukin-2 receptor; IL-6, interleukin-6; IL-8, interleukin-8; IL-10, interleukin-10; TNF-α, tumor necrosis factor-α*.

### AEC in Predicting ICU Transfer Among Elderly COVID-19 Patients

**Figure 3:**
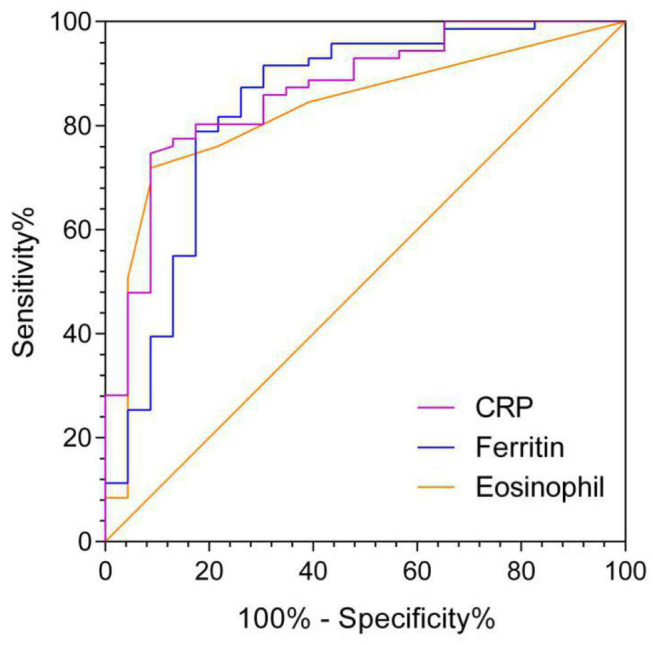
ROC of variables in predicting ICU transfer among elderly COVID-19 patients on admission.

The AUC of AEC on admission in predicting ICU transfer among elderly COVID-19 patients was 0.828 (95% CI, 0.732–0.923), whereas the AUCs of CRP and ferritin were 0.867 (95% CI, 0.784–0.951) and 0.838 (95% CI, 0.730–0.947), respectively, with no significant difference in the pairwise comparison of ROC curves (*P* > 0.05; Figure 3 and Table 7). The best cut-off value of AEC was 25 cells/μl, yielding a sensitivity of 91%, a specificity of 71%, a positive predicted value of 51%, and a negative predicted value of 98%, respectively.

**Table 7:** The AUC of variables in predicting ICU transfer among elderly COVID-19 patients on admission.

**Variables**	**AUC**	**SE**	**95% CI**
AEC	0.828	0.0488	0.732–0.923
CRP	0.867	0.0425	0.784–0.951
Ferritin	0.838	0.0554	0.730–0.947

*AUC, area under the curve; ICU, intensive care unit; COVID-19, coronavirus disease 2019; SE, standard error; CI, confidence interval; C-reactive protein*.

## Discussion

We conducted the present study to evaluate the association between eosinopenia and ICU transfer prediction of elderly COVID-19 patients from general isolation wards. Our results showed that the eosinopenia group had a significantly higher ICU transfer rate in the eosinopenia group than in the non-eosinopenia group (51 vs. 9%, *P* < 0.001). In univariate analysis and multivariate analysis, the strong inverse association between eosinopenia and ICU transfer of elderly COVID-19 patients remained significant. Moreover, the AUC of AEC in predicting ICU transfer for elderly COVID-19 patients was equivalent to that of CRP and ferritin. Collectively, AEC could be a valid predictive marker of ICU transfer for elderly COVID-19 patients from general isolation wards.

The mechanism of eosinopenia has not been completely understood in COVID-19. Eosinophils were observed in the infiltrating immune cells of the lungs as revealed by minimal autopsy of dead COVID-19 patients (16). It is assumed that eosinopenia is the redistribution result of circulating eosinophils to the infection locus due to the chemotactic effects of increased cytokines (17). Cytokine storm was observed in severe COVID-19 patients with high levels of various serum cytokines, such as IL-2, IL-7, IL-10, TNF-α, granulocyte-colony stimulating factor (G-CSF), interferon gamma-induced protein 10 (IP-10; CXCL10), and monocyte chemotactic protein-1 (MCP-1) (18–21). We demonstrated that concentrations of classic inflammation indicators, including CRP, ferritin, and several serum cytokines (IL-2R, IL-6, IL-8, IL-10, and TNF-α), were significantly higher in the eosinopenia group than in the non-eosinopenia group. Surprisingly, AEC was zero in more than half (61%) of the patients who eventually transferred to the ICU in this study. This phenomenon described by Shaaban et al. (9) was called “the almost zero eosinophil effect,” probably because of the overwhelming effects of cytokine storm caused by severe infection. The above evidences suggest that eosinopenia in COVID-19 patients may be due to cytokine storm.

The association between AEC and COVID-19 has been explored by limited studies. Eosinopenia was observed in COVID-19 patients with a high percentage up to 47–66% (22, 23). Moreover, compared with mild COVID-19 patients, severe patients had much lower AEC (24–26). Similarly, eosinopenia was detected in 37% of our COVID-19 patients, which was highly correlated with ICU transfer as revealed by multivariate analysis. Therefore, it is suggested that AEC could be a valid predictive marker for severe elderly COVID-19 patients.

Additionally, our results showed that the AUC of AEC on admission for predicting ICU transfer among elderly COVID-19 patients was 0.828, nearly equivalent to that of CRP and ferritin. Compared with the expensive and time-consuming CRP and ferritin, AEC is a cost-effective, convenient, and fast predictive marker, which is helpful for health care facilities to allocate limited ICU resources more reasonably.

Our study has some limitations. Firstly, it is a retrospective study that would provide limited evidence. Secondly, it is a single-center study including only those patients older than 60 years old, but not patients of all ages. Thirdly, the evaluation of AEC is in a single time point on admission without subsequent dynamic changes due to systemic corticosteroids treatment. Hence, a multicenter study with a larger cohort is required for further validation of our conclusion.

## Conclusions

We demonstrated that AEC on general admission is a valid predictive marker for ICU transfer among elderly COVID-19 patients from general isolation wards and, therefore, can help case triage and optimize ICU utilization. Additionally, AEC is cost-effective, convenient, and fast compared with the expensive and time-consuming CRP and ferritin, making it an appropriate alternative for health care facilities with limited ICU capacity.

## Data Availability Statement

The original contributions generated in the study are included in the article/supplementary materials, further inquiries can be directed to the corresponding author.

## Ethics Statement

The studies involving human participants were reviewed and approved by Tongji Hospital, Tongji Medical College, Huazhong University of Science and Technology. The patients/participants provided their written informed consent to participate in this study.

## Author Contributions

JH and ZZ designed the study and drafted the manuscript. GA and LC collected and summarized all epidemiological and clinical data. SL, XZ, CZ, and CG contributed to the statistical analysis. DL and ZZ revised the final manuscript. All authors contributed to the article and approved the submitted version.

## Conflict of Interest

The authors declare that the research was conducted in the absence of any commercial or financial relationships that could be construed as a potential conflict of interest.

## Abbreviations

AEC: absolute eosinophil count
COVID-19: coronavirus disease 2019
SARS-CoV-2: severe acute respiratory syndrome coronavirus 2
ICU: intensive care unit
CRP: C-reactive protein
Hs-cTnI: hypersensitive cardiac troponin I
NT-ProBNP: NT-ProB-type natriuretic peptide
IL-2R: interleukin-2 receptor
IL-6: interleukin-6
IL-8: interleukin-8
IL-10: interleukin-10
IQR: interquartile range
TNF-α: tumor necrosis factor-α
PT: prothrombin time
APTT: activated partial thromboplastin time
GFR: glomerular filtration rate
WBC: white blood cell
ALT: alanine aminotransferase
AST: aspartate aminotransferase
ROC: receiver operating characteristics
AUC: area under the curve
OR: odds ratio
95% CI: 95% confidence interval
PPV: positive predicted value
NPV: negative predicted value
G-CSF: granulocyte-colony stimulating factor
IP-10: interferon gamma-induced protein 10
MCP-1: monocyte chemotactic protein-1
SARS: systemic inflammatory response syndrome.
